# Anhedonia and Anxious Arousal Are Associated With Distinct Expectations About the Statistics of a Volatile Environment

**DOI:** 10.1016/j.bpsgos.2026.100736

**Published:** 2026-04-21

**Authors:** Jessica M. Duda, Tyrone D. Cannon, Samuel Paskewitz, William C. Palmer, Praveen Suthaharan, Joshua G. Kenney, Philip R. Corlett, Jutta Joormann

**Affiliations:** a Department of Psychology, Yale University, New Haven, Connecticut; b Department of Psychiatry, Yale University, New Haven, Connecticut

**Keywords:** Anhedonia, Anxiety, Computational modeling, Hierarchical Bayesian inference, Uncertainty, Volatility

## Abstract

**Background:**

The brain is believed to construct a generative model of the environment, updated via differences between predictions and incoming sensory evidence (i.e., prediction errors). Uncertainty can arise at multiple levels, including when estimating the environment’s causal structure (stochasticity; first order), the volatility of this causal structure (second order), and volatility of this volatility (meta-volatility; higher order). Altered estimation of first-order uncertainty is implicated in anxiety and depression, including inflated certainty about the probability of negative events, but the role of second- and higher-order uncertainty estimation in internalizing disorders is less clear.

**Methods:**

In a crowdsourced community sample (*N* = 287), we examined associations between depressive and anxiety symptoms and beliefs about volatility derived with a hierarchical Bayesian model of inference applied to a rewarding and aversive probabilistic reversal-learning task.

**Results:**

Anxious arousal was linked to elevated initial expectations of volatility in an aversive context and greater adjustment of behavior after avoiding a loss. Meanwhile, anhedonia was associated with reduced initial expectations of volatility, relatively less stable volatility beliefs in reward versus loss contexts early in learning (indexed by higher meta-volatility), and reduced exploratory behavior in a loss environment.

**Conclusions:**

Together, the results indicate distinct belief signatures of anxiety and depression in a volatile environment. Elevated expectations of the volatility of aversive contexts could increase hypervigilance, a key anxiety symptom, whereas a belief that the environment is stagnant could increase hopelessness and anhedonia, markers of depression. Distinct alterations in estimating volatility could present candidate mechanisms and treatment targets for anxiety and depression.

## Plain Language Summary

To maintain accurate beliefs in a changing world, people must effectively estimate when conditions will shift. Misjudging how often environmental changes occur may produce beliefs that are either overly rigid or flexible, which could contribute to mental health problems. In this study, we showed that anhedonia (a key depressive symptom) may involve an expectation of more stagnant or fixed conditions, while anxious arousal may be linked to expectations of more frequent environmental changes, especially in negative contexts. Differences in estimating how rapidly the world changes could play a role in anxiety and depression and be a target for future psychotherapy.

The causes of events are thought to be inferred by combining sensory evidence with prior beliefs about the environment (1). Uncertainty arises when estimating the environment’s causal structure (stochasticity; first order), which is compounded by the fact that this causal structure is often changing (volatility; second- and higher order) (2, 3, 4, 5). Biases in estimating each form of uncertainty can produce inaccurate beliefs about environmental statistics, which may contribute to a range of psychopathology [e.g., see (6,7)]. Biased first-order uncertainty estimation is implicated in anxiety and depression (8, 9, 10), and overestimating the probability of negative events is a primary target of cognitive behavioral interventions (11,12). Unfortunately, existing therapies are only partially effective in most patients (13), highlighting the need for new modifiable treatment targets. Here, we investigated the associations between anxiety and depressive symptoms and second- and higher-order uncertainty estimation, an understudied candidate mechanism of internalizing disorders.

### Uncertainty Estimation in Anxiety and Depression

Beliefs about uncertainty are widely implicated in anxiety disorders, including intolerance of uncertainty (14) and inflated estimates of the probability of negative outcomes held with high certainty (9,15, 16, 17). According to theoretical work rooted in Bayesian decision theory, individuals with anxiety form wide-ranging estimates of the probability of negative events with imprecise upper limits, overutilizing the upper ranges of these estimates to avoid catastrophic outcomes (8,18). Heightened estimates of environmental volatility may contribute to these negative expectations, as negative events could suddenly become more probable if environmental conditions change (8). These elevated volatility estimates could reflect preexisting biases in second-order uncertainty estimation or a misattribution of first-order uncertainty (stochasticity) as second-order uncertainty (volatility) (4). Few studies have empirically examined volatility and stochasticity estimation in anxiety disorders, although elevated volatility priors have been linked to worry in schizophrenia (19), state anxiety (20), trait anxiety in healthy control participants (21), and trait anxiety in bipolar disorder (22). Individuals with anxiety also show impaired adjustment of learning rates to volatility (23, 24, 25, 26, 27, 28), theorized to reflect elevated volatility priors (8). However, research is needed that directly examines how anxiety alters volatility estimation across the spectrum of severity, especially comparing rewarding versus aversive environments, given evidence of asymmetric recruitment of neural systems that support volatility estimation by valence (28) and specific alterations in beliefs about negative events in anxiety (29, 30, 31, 32).

Depression also involves elevated estimates of the probability of negative outcomes and is further characterized by diminished anticipation of positive events (33, 34, 35, 36). Expecting outcomes to be unpredictable (first-order uncertainty) may bias encoding toward negative events and slow associative learning in depression (6,37). Low estimates of volatility (second- and higher-order uncertainty) could also slow associative learning, as a given cue is less predictive of change in a stagnant environment (4). There are only a few studies on volatility estimation in depression. Reduced adjustment of learning to volatility has been associated with general distress (38) and depressive symptoms [(39,40) but see (28)], which could be driven by altered uncertainty estimates. Studies are needed to test this possibility, including comparing beliefs by valence given diverging decision making about reward versus loss in depression (30, 31, 32).

### The Hierarchical Gaussian Filter

**Figure 1:**
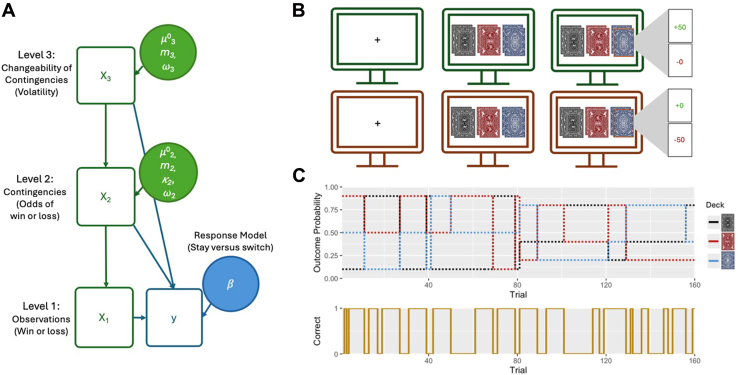
Deriving volatility estimates from reversal learning. **(A)** Three-level mean-reverting hierarchical Gaussian filter (54). Level 1 (x_1_) represents observations of win versus no win (rewarding version) or loss versus no loss (aversive version). Level 2 (x_2_) represents an agent’s expectations of the outcome probabilities associated with each card deck. *μ*^0^_2_ and *m*_2_ are the prior and equilibrium expectations, respectively, of the outcome probabilities. *κ*_2_ is a coupling parameter between levels 3 and 2, and *ω*_2_ is the rate of adjustment of reward or loss expectations (tonic volatility). Level 3 (x_3_) represents expectations of the changeability of the contingencies associated with each deck. *μ*^0^_3_ and *m*_3_ are participants’ prior and equilibrium volatility estimates, respectively, and *ω*_3_ is the rate of adjustment of volatility expectations (meta-volatility). Agents’ responses (y) are determined by a softmax μ_3_ decision model with inverse decision temperature *β*. **(B)** Sample trial from the reward version (top) and aversive version (bottom) of a probabilistic reversal-learning paradigm. On each trial of the reward version, participants could win 50 points or lose 0 points, and on each trial of the loss version, they could win 0 points or lose 50 points. **(C)** Example outcome contingency schedule (top) based on whether an agent selected the best deck on each trial (bottom). In the first 80 trials, decks were associated with a hidden 90%, 50%, or 10% probability of a reward (reward version) or no loss (aversive version), and in the second 80 trials, decks were associated with an 80%, 40%, or 20% probability of reward (or no loss). Hidden deck probabilities switched every 40 trials (performance independent) and after selecting the best deck in 9 of the last 10 trials (performance dependent).

The hierarchical Gaussian filter (HGF), a formal algorithm implementing hierarchical Bayesian inference, is increasingly used to study beliefs about volatility in psychopathology (19,21,22,41, 42, 43, 44). In contrast with traditional reinforcement learning models, the HGF directly derives parameters indexing beliefs about environmental statistics. It includes a multilevel perceptual model, representing beliefs about the environment, and an observation model, linking beliefs to behavior (Figure 1A). Third-level parameters estimate beliefs about volatility (e.g., how often win or loss probabilities shift), including an initial volatility estimate (μ^0^_3_), equilibrium volatility expectation toward which the volatility estimate drifts (*m*_3_), and meta-volatility learning rate (*ω*_3_), with a higher value indicating greater updating of volatility beliefs. The model is well suited for examining second- and higher-order uncertainty estimates in anxiety and depression.

### The Current Study

In this study, we examined associations between anxiety and depressive symptoms and HGF-derived volatility estimates from rewarding and loss versions of a probabilistic reversal-learning (PRL) paradigm. Switch behavior and U-values [a measure of choice stochasticity (45)] were examined as possible behavioral correlates of volatility beliefs (41). Given considerable symptom overlap between internalizing disorders (46), dimensional scales were chosen that separate unique and overlapping anxiety and depressive symptoms (47). We posed the following hypotheses:

1. First, anxious arousal will be associated with higher but inflexible volatility estimates, especially in an aversive environment, with 1) higher switch rates and U-values, 2) greater expectations of volatility (μ^0^_3_ and *m*_3_), and 3) reduced adjustment of volatility expectations (*ω*_3_), given a hypothesized role of elevated volatility estimates in reduced adaptation of learning to volatility in aversive contexts in anxiety (8,24,27).
2. Anhedonia will be associated with lower volatility estimates, especially in a rewarding environment, with 1) lower switch rates and U-values and 2) reduced expectations of volatility (μ^0^_3_ and *m*_3_), consistent with evidence of slowed associative reward learning in depression (37,39,48). Given evidence of opposing associations between psychopathology and parameters capturing volatility expectations versus meta-volatility (41), anhedonia may also be associated with 3) greater adjustment of volatility expectations (*ω*_3_).

We did not hypothesize consistent associations between general distress and volatility estimation, given offsetting predictions for the anxiety- and depression-specific dimensions.

## Methods and Materials

### Sample and Procedure

A sample of 287 adults in the United States was recruited via Prolific and completed clinical questionnaires and valence-specific PRL paradigms (41). A crowdsourced sample was chosen to 1) facilitate iterative piloting, 2) reach a larger sample than in-person studies, and 3) recruit participants with a wide range of symptom severity (Sample Characteristics). Prolific samples have been shown to exhibit data quality superior to that of undergraduate, Qualtrics, and MTurk samples (49). A final sample of 223 remained after quality controls (Supplement).

### Questionnaires

#### Mood and Anxiety Symptom Questionnaire–30-Item Adaptation

The 30-item adaptation of the Mood and Anxiety Symptom Questionnaire (MASQ-30) comprises 30 items assessing general distress, anxious arousal (anxiety-specific physiological symptoms), and anhedonia (depression-specific loss of interest), reflecting the tripartite model of anxiety and depression (47). The scale was chosen due to the relative independence of the 3 subscales (50,51), contrasting with the high symptom overlap of many anxiety and depression scales [e.g., (46)]. The MASQ-30 demonstrates strong psychometric properties (51,52).

#### Revised Green Paranoid Thoughts Scale–Ideas of Persecution Subscale

Given findings of altered volatility estimation in schizophrenia spectrum disorders (19,41), the 10-item Revised Green Paranoid Thoughts Scale (R-GPTS) part B was included to query persecutory ideation. The R-GPTS shows strong psychometric properties and reliability across symptom severity levels (53).

### PRL Paradigm

Participants completed two 160-trial runs of a 3-option PRL paradigm (Figure 1B) (41). To separate decision making in rewarding versus aversive environments, each person completed counterbalanced versions with reward (+50 vs. 0 points on each trial, 0 starting points) and loss (−50 vs. 0 points, 8000 starting points). On each trial, participants selected a card deck to receive a reward (or avoid a loss) and were told that the contingencies might change. Unbeknownst to participants, contingencies switched after selecting the deck with the highest probability of reward/lowest probability of loss in 9 of 10 trials and independently of performance after every 40 trials (Figure 1C). Deck reward (no-loss) probabilities were 90%, 50%, and 10% and 80%, 40%, and 20% in the first and second blocks, respectively. Past work varying these contingencies found the current version with increasing uncertainty to be most clinically relevant (41).

### Analyses

#### Behavioral Metrics

Switch rates and U-values are thought to be sensitive to volatility expectations, as greater exploratory behavior is optimal under volatility (5,41). Win-switch rates were the proportion of win trials (gain version) or no-lose trials (loss version) after which participants selected a new deck. Lose-switch rates were the proportion of lose trials (loss version) or no-win trials (gain version) followed by a new deck selection. U-values were computed as a measure of choice stochasticity:

(1) $$U\text{-value}=-\sum_{i=1}^{\beta }\frac{\log\left(\alpha_{i}\right)\times \alpha_{i}}{\log\left(\beta \right)}\text{,}$$

where β represents the number of decks, and α is the proportion of times that deck i with a given probability of reward/no loss was selected (45). A higher U-value indicates more variability in choice behavior.

#### Computational Modeling: Hierarchical Gaussian Filter

A mean-reverting 3-level HGF was selected for the perceptual model (43,54,55) (Table S1). A 3-level model has been shown to best explain behavior on the combined-valence task (41) and is well suited to deriving volatility estimates. Given evidence of differences in clinical associations by task block in the combined-valence task (41,43), separate free parameters were estimated by block within a single model without reinitializing the trajectories, with a single μ^0^_3_ reflecting initial volatility expectations. The split-block model showed a superior fit to a model with parameters estimated across the full task (Supplement). A softmax μ_3_ decision model was applied (41,43,54). The HGF Toolbox version 7.1 is publicly available from the Translational Algorithms for Psychiatry-Advancing Science repository (56,57).

#### Computational Modeling: Q-Learning Model

A Q-learning model with separate learning rates for positive and negative prediction errors was also tested to be consistent with past work (41). Learning rate and inverse temperature parameters were not significantly related to internalizing symptoms (Supplement).

#### Statistics

Linear mixed-effects models were run with the *nlme* package in R (58,59) predicting switch behavior and third-level volatility estimates, with fixed effects of clinical symptoms (anhedonia, anxious arousal, or general distress), task valence (gain vs. loss), task block, their interactions, and a participant-specific random intercept. Given well-replicated associations between schizophrenia spectrum symptoms and task behavior (19,41, 42, 43,55) and the potential for paranoia to drive anxiety, persecutory ideation was also included in the models.

Given skewness in win-switch rates, binomial generalized linear mixed models (GLMMs) were run predicting win-switch counts to confirm findings. Sensitivity analyses with anxious arousal and anhedonia added into a single model were also run for all significant findings, with results unchanged. Correlations between demographics and each outcome variable were tested, and variables showing significant associations with volatility estimation or behavior were added as covariates to the relevant models. See the Supplement for more information about sensitivity analyses.

## Results

### Sample Characteristics

See Table 1 for sample demographic characteristics. The sample reported meaningful variability in MASQ anhedonia (mean = 34.4, SD = 10.1, range = 12–50), anxious arousal (mean = 16.1, SD = 7.0, range = 10–46), and general distress (mean = 22.0, SD = 10.0, range = 10–49) scores. Average symptom severity was near or above cutoffs differentiating outpatients with mood and anxiety disorders from the general population (60). Lifetime anxiety and depression diagnoses were reported by 28.3% and 26.5% of the sample, respectively (Table S2). General distress was strongly correlated with both anxious arousal, (*r*_221_ = 0.61, *p* < .001) and anhedonia (*r*_221_ = 0.61, *p* < .001), supporting a shared negative affect dimension (47), while anxious arousal and anhedonia were only weakly correlated (*r*_221_ = 0.23, *p* < .001).

**Table 1:** Demographics of the Final Sample (*N* = 223)

Characteristic	Mean (SD) or *n* (%)
Age, Years	41.5 (12.7)
Annual Income, $K	84.4 (60.8)
Gender Identity	
Agender	1 (0.4%)
Female	102 (51.1%)
Male	114 (45.7%)
Nonbinary	6 (2.7%)
Race	
Asian or Asian American	17 (7.4%)
Black or African American	35 (15.2%)
Native American or Alaska Native	2 (0.9%)
Native Hawaiian or Pacific Islander	1 (0.4%)
White or European American	174 (75.3%)
Not disclosed	2 (0.9%)
Ethnicity	
Hispanic or Latine	23 (10.3%)
Middle Eastern or North African	5 (2.2%)
Neither	195 (87.4%)
Education	
High school graduate	30 (13.5%)
Some college	43 (19.3%)
Vocational training	6 (2.7%)
Associate degree	18 (8.1%)
Bachelor’s degree	92 (41.3%)
Graduate degree	34 (15.2%)

Race category includes multiracial participants and thus may sum to greater than the sample size.

### Task Validation

The task showed adequate convergent validity, with accuracy and behavior aligning with both the established combined-valence version (41) and across the new reward and loss versions (Supplement). Previously identified associations between paranoia, win-switch rate, and equilibrium volatility expectations (*m*_3_) (19,41,43) were largely replicated with the new task (see the Supplement for further paranoia findings). Internalizing symptoms were not significantly related to task performance (Table S3) or consistently associated with the second-level HGF parameters (Table S4).

### Parameter Recovery and Optimization

Parameter recovery for the third-level HGF parameters was moderate for μ^0^_3,_ strong for *m*_3,_ and weak for ω_3_ (Supplement). The model predicted participant choices with good accuracy (74.1% of trials) (Figure S1). A grid search for μ^0^_3_ and ω_3_ ranges that optimized task performance indicated that lower μ^0^_3_ and higher ω_3_ tended to be more optimal (Figure S2).

### Anxious Arousal

**Figure 2:**
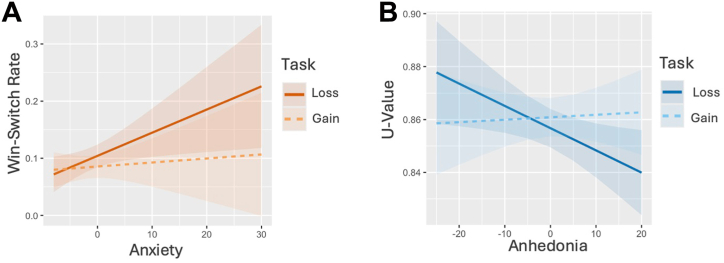
Anxious arousal and anhedonia are associated with greater and reduced exploratory behavior, respectively, in an aversive context. Mixed-effects models with task block, task valence (gain vs. loss), and a participant-specific random intercept, controlling for paranoia, predicting **(A)** win-switch rates by anxious arousal and **(B)** U-values by anhedonia. Anxious arousal and anhedonia were measured with the 30-item adaptation of the Mood and Anxiety Symptom Questionnaire (52) and mean centered prior to analyses. Anxious arousal interacted with task valence to predict a greater win-switch rate in the loss vs. gain versions of the probabilistic reversal-learning task (*t*_657_ = 2.80, *p* = .005). Anhedonia interacted with task valence to predict lower U-values in the loss vs. gain versions (*t*_657_ = −2.12, *p* = .035). Anxious arousal and its interaction with valence were not significantly associated with lose-switch rate (*p*s > .519) or U-values (*p*s > .222). Anhedonia was not significantly related to switch behavior (*p*s > .166). See Figure S3 for visualizations of anxious arousal, anhedonia, and general distress with all switch rates and U-values.

**Figure 3:**
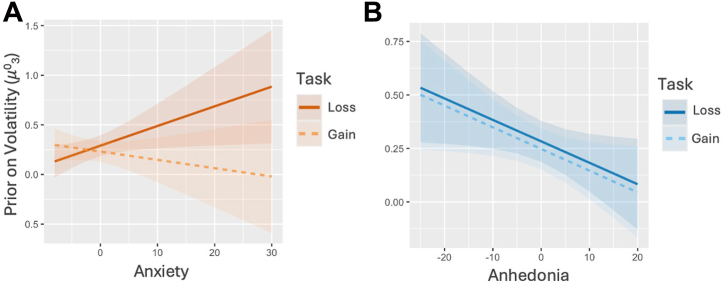
Anhedonia and anxious arousal are associated with distinct prior beliefs about volatility. Anxious arousal and anhedonia predicting prior beliefs about volatility (*μ*^0^_3_) derived from a 3-level hierarchical Gaussian filter fit to behavior on 2 probabilistic reversal-learning tasks. Clinical symptoms were measured with the 30-item adaptation of the Mood and Anxiety Symptom Questionnaire (52). Depicted results are from linear mixed-effects models predicting *μ*^0^_3_ by task valence and **(A)** anxious arousal or **(B)** anhedonia, controlling for paranoia, with a random intercept for participant. Anxious arousal interacted with valence to predict a greater initial expectation of volatility in the loss vs. gain versions of the task (*t*_219_ = 2.24, *p* = .026), whereas anhedonia predicted a reduced initial expectation of volatility (*μ*^0^_3_) (*t*_219_ = −2.90, *p* = .004), regardless of task valence (*p* = .987). See Figure S4 for a visualization of *μ*^0^_3_ findings with the general distress dimension.

Partially supporting our hypothesis that anxiety would be associated with higher volatility estimates and corresponding behavior in aversive conditions (hypothesis 1), anxious arousal interacted with task valence to predict greater win-switch rates in the loss task (i.e., no-lose switch) versus gain task after accounting for paranoia (*t*_657_ = 2.80, *p* = .005, η_p_^2^ = 0.01 (Figure 2A; see the Supplement for converging findings with a binomial GLMM). Contrasting with our hypotheses, anxiety and its interaction with valence were not significantly associated with lose-switch behavior or U-values (*p*s ≥ .222). In terms of model-based analyses, a significant interaction between anxious arousal and task valence was associated with higher prior volatility estimates (μ^0^_3_) in the loss versus gain tasks (*t*_219_ = 2.24, *p* = .026, η_p_^2^ = 0.02), consistent with our hypotheses (Figure 3A). Counter to our hypotheses, anxiety and its interaction with valence were not significantly associated with equilibrium volatility beliefs (*m*_3_) (*p*s > .510) or meta-volatility (ω_3_) (*p*s > .346).

### Anhedonia

Consistent with hypotheses that anhedonia would be associated with reduced volatility estimates and exploratory behavior (hypothesis 2), anhedonia interacted with valence to predict lower U-values (*t*_657_ = −2.12, *p* = .035, η_p_^2^ = 0.01). The interaction was in a direction opposite to that hypothesized, with anhedonia predicting lower U-values in the loss versus gain tasks (Figure 2B). Anhedonia and its interactions with task valence predicting overall switch rates were not significant (*p*s > .166), although a negative association between anhedonia and both trial-level and total win-switch counts emerged regardless of valence in a binomial GLMM to account for skewness (Supplement). In our model-based analyses, anhedonia predicted a lower prior expectation of volatility (μ^0^_3_) (*t*_219_ = −2.90, *p* = .004, η_p_^2^ = 0.04) with no valence interaction (*p* = .987), in contrast to our hypotheses (Figure 3B). Anhedonia and the equilibrium expectation of volatility (*m*_3_) were only marginally associated (*t*_219_ = −1.72, *p* = .087), with no significant valence interaction (*p* = .721).

Consistent with hypotheses that anhedonia would increase adjustment of volatility expectations, anhedonia predicted a higher meta-volatility learning rate, ω_3_ (*t*_219_ = 2.17, *p* = .031, η_p_^2^ = 0.02), but did not significantly interact with valence (*p* = .553). However, an anhedonia × valence × and task block interaction effect emerged (*t*_657_ = −2.63, *p* = .009, η_p_^2^ = 0.01). As an exploratory follow-up analysis, mixed-effects models run separately by block revealed a significant anhedonia × task valence interaction in the first half of the task (*t*_219_ = 2.26, *p* = .025, η_p_^2^ = 0.02) but not the second half (*p* = .118). Specifically, anhedonia was linked to greater adjustment of volatility beliefs in the first half of the gain condition (*t*_219_ = 2.72, *p* = .007, η_p_^2^ = 0.03) but not the first half of the loss condition (*p* = .978), partially supporting our hypotheses. Despite higher meta-volatility, anhedonia was not significantly associated with a net increase or decrease in volatility expectations (μ_3_) over the block (*p* = .989).

### General Distress

Associations between general distress, task behavior, and volatility estimates were inconsistent. General distress and its interactions with valence were not significantly related to switch behavior (*p*s > .091), U-values (*p*s > .386), or meta-volatility (ω_3_) (*p*s > .095) (Figure S3). General distress predicted lower equilibrium volatility expectations (*m*_3_) (*t*_219_ = −2.28, *p* = .024, η_p_^2^ = 0.001) and lower volatility priors (μ^0^_3_) (*t*_219_ = −2.20, *p* = .029, η_p_^2^ = 0.01), especially in the gain condition (*t*_219_ = −2.00, *p* = .047, η_p_^2^ = 0.02) (Figure S4), but these effects did not reach significance when paranoia was removed from the model.

## Discussion

### Summary

In this study, we examined how anxiety and depressive symptoms related to behavior and uncertainty estimation in volatile rewarding and aversive contexts. Despite considerable symptom overlap, anxiety and depression can be distinguished by physiological hyperarousal and low positive affect, respectively (47); we applied self-report measures designed to isolate these dimensions. We found that internalizing symptoms were significantly associated with initial expectations of volatility (μ^0^_3_), but not equilibrium volatility expectations (*m*_3_), indicating preexisting biases about environmental statistics. Anxious arousal was associated with an elevated prior volatility estimate for aversive contexts, accompanied by more switching after safe outcomes. Meanwhile, anhedonia was associated with a lower prior volatility estimate across contexts, greater early flexibility of these expectations (ω_3_) in the gain task (indicating comparatively greater initial certainty about volatility estimates in a loss vs. reward environment), and lower choice stochasticity (U-value) in an aversive environment. Together, these results indicate that anhedonia appears to involve a bias toward expecting less environmental change, with less initial certainty about these beliefs in rewarding versus aversive contexts, while anxious arousal involves expectations of greater volatility in aversive environments.

### Anxious Arousal Predicts Higher Initial Volatility Estimates

Anxious arousal was associated with more choice switching after a safe outcome, consistent with past work showing higher switch rates in anxiety (26,61). By separating switch behavior by valence, we revealed greater switching specifically after avoiding a loss, thereby adding to evidence of valence-specific alterations in decision making in anxiety (29, 30, 31, 32). At the first level, continued exploratory behavior after no-lose outcomes could reflect a distrust of evidence of safety due to threat-related priors (8) or anxious avoidance of perceived possible future negative outcomes (62). Misclassification of first-order uncertainty as second-order uncertainty (misattributing noise to volatility) could also spur exploratory behavior (4), although we did not find consistent associations with *ω*_2,_ which may be sensitive to noise estimates (Table S4). Alternatively, our findings of increased volatility priors in an aversive environment in anxiety may also explain greater switching after safe choices, as hidden loss probabilities are more likely to have shifted between trials in a volatile environment (5). Past work found reduced adaptation of learning to volatility in aversive contexts in anxiety (24,27,28), theorized to reflect high volatility priors (8). However, previous evidence of elevated volatility estimation in anxiety has come from samples of individuals with schizophrenia or bipolar disorder or healthy individuals with no psychiatric history (19,21,22,63) and has not compared beliefs by valence to our knowledge. The current study extends these findings by showing that anxious arousal across a spectrum of severity is linked to a belief that aversive environments are volatile, making it appear adaptive to avoid previously safe choices.

### Anhedonia Predicts Lower Initial Volatility Estimates

In contrast, anhedonia was linked to a perception of the environment as less changeable (reduced prior on volatility), regardless of valence. These volatility expectations were more rapidly adjusted (higher meta-volatility learning rate) early in the gain task, which could indicate less initial certainty about the volatility of a reward environment and comparatively greater initial certainty about the volatility of a loss environment. Behavior was more deterministic in the loss condition (lower U-value), which may reflect motivational deficits in aversive contexts (low U-values indicate less differentiation in the valuation of options) or reduced volatility expectations (41). A binomial GLMM predicting switch behavior revealed further evidence of reduced exploratory behavior (specifically after wins and no-losses) in anhedonia, converging with computational findings of reduced volatility expectations across valences.

Evidence of reduced volatility expectations in depression is consistent with work connecting anhedonia to elevated estimates of environmental unpredictability (6). Expecting the world to be both unpredictable and unchanging may appear contradictory, but both should reduce learning rates, as cues are less predictive of outcomes in a noisy environment and less predictive of change in a stable environment (4). Together, depression may involve an expectation of unpredictable, but stagnant, environmental conditions. Paired with established negative interpretation biases (64), expecting stagnant conditions may constrain behavior, reducing exploration (5), behavioral flexibility (65), and efforts to change the environment (66)—key markers of motivational anhedonia. Our results suggest that a person with anhedonia may initially be relatively more certain about stagnant conditions in an aversive environment and uncertain about what will happen in a positive environment, which could further dampen anticipatory pleasure and contribute to hopelessness.

### Extension to Comorbid Anxiety and Depression

Although our findings are largely specific to anxious arousal and anhedonia, high rates of comorbid anxious depression (67) complicate interpretation. Several explanations are possible. First, anxious depression could involve negative affect alongside anxious arousal or anhedonia, without anxious arousal and anhedonia typically co-occurring. This interpretation is consistent with the tripartite model of anxiety and depression (47) and with our findings of strong correlations between general distress and both anxious arousal and anhedonia (which in turn were only weakly correlated). Second, anxious depression could combine the volatility belief signatures of anxious arousal and anhedonia, leading to reduced volatility expectations in reward conditions and offsetting expectations (elevated in anxiety, reduced in anhedonia) under loss conditions. Our general distress dimension was linked specifically to lower volatility expectations under reward conditions, which is consistent with findings of reduced learning from better-than-expected outcomes under volatility in general distress (38). Third, anxious depression may reflect primarily general distress alone, without corresponding changes in volatility beliefs, which would align with our inconsistent findings with the general distress dimension. Finally, volatility expectations could parallel state-dependent fluctuations in anxious arousal or anhedonia (68), regardless of trait-level comorbidities. Clinical samples with anxious depression versus anxiety or depression alone and longitudinal data will be needed to clarify these mechanisms.

### Limitations

Although this study provides insights about the role of higher-order uncertainty estimation in anxiety and depression, several limitations should be considered. First, generalizability to clinical populations remains to be tested, despite the range of clinical symptom severity in this sample (including average anhedonia above established cutoffs) (60). Second, switch rates and U-values may index volatility expectations (41), but claims about underlying cognitive processes remain speculative. Careful quality control and a lack of significant links between internalizing symptoms and task performance are encouraging, but we cannot fully rule out effects of engagement and motivation on results. Third, while the results intuitively extend to real-world behavior, future work should test these relationships directly, such as with experience sampling methods. Fourth, control of testing conditions online is limited, and replication in the laboratory is recommended. Fifth, while model recovery was largely adequate, correlations between the recovered and actual ω_3_ were poor to modest, and the range of values for the recovered μ^0^_3_ was truncated. This pattern is consistent with past work applying the HGF to study psychopathology (41) and is a known issue with recovering HGF parameters at the top of the hierarchy (69). To establish parameter stability and confirm our findings, replication in a separate sample and with longitudinal data is warranted. Finally, it remains unclear whether our findings represent state or trait markers of anxiety and anhedonia. For example, hyperarousal may cause greater sensitivity to environmental changes, inflating volatility beliefs, or conversely, expecting volatility may increase attention to evidence of change, leading to hyperarousal. Longitudinal research could clarify this directionality.

### Contributions and Future Directions

Despite these limitations, this work addresses several gaps in the literature. Previous studies examined adaptation of learning to volatility in anxiety [e.g., (24,27)] and depression [e.g., (40)], but the role of higher-order uncertainty estimation remained less clear. Our approach of separating uncertainty estimation by valence revealed altered volatility estimates that were only apparent in an aversive or rewarding environment. These findings are consistent with research showing asymmetric recruitment of neural systems that control responsiveness to volatility across positive and negative contexts [e.g., norepinephrine (28)] and with findings of valence-specific learning alterations in internalizing disorders (31,32,70,71).

Our use of a dimensional measure that isolated shared versus unique symptoms of anxiety and depression also revealed distinct volatility belief signatures that might have otherwise been hidden. Many self-report internalizing measures (e.g., Beck Depression Inventory, State-Trait Anxiety Inventory) are highly overlapping and thought to capture general distress (46). Given our inconsistent findings with the general distress dimension, applying these measures might have masked depression- and anxiety-specific associations.

A strength of computational methods is their ability to reveal latent cognitive processes, which can be linked to neural circuitry to identify neurobiological mechanisms of psychopathology. The anterior cingulate cortex (ACC) and dorsolateral prefrontal cortex (dlPFC) are implicated in estimating volatility (2,27,72,73) and could contribute to altered volatility estimation in internalizing disorders. The ACC and dlPFC are thought to show altered function during learning and decision making in anxiety (21,27,74) and depression (75, 76, 77), and dlPFC transcranial stimulation has been found to normalize adaptation of learning to volatility in depression (40). Future studies should examine how altered volatility estimates relate to these neural correlates in internalizing disorders.

A possible clinical implication of this work is targeting second- and higher-order uncertainty estimation with novel intervention. Existing cognitive behavioral and attention bias modification treatments largely address first-order uncertainty estimation, including challenging negative beliefs and training attention toward positive information (78,79). Treatments could draw on these approaches to shift volatility estimates to better match the environment, encouraging greater comfort in the stability of the environment in anxiety and hope for change in depression.

## Supplementary Material

Supplemental Methods, Results, Figures S1–S5, and Tables S1–S4

Key Resources Table
