# Supplementary material for: Anhedonia and Anxious Arousal Are Associated With Distinct Expectations About the Statistics of a Volatile Environment
Source: Biol Psychiatry Glob Open Sci. 2026 Apr 21;6(4):100736. doi: 10.1016/j.bpsgos.2026.100736 (PMC13260089; doi:10.1016/j.bpsgos.2026.100736)
Supplement: Supplemental Methods, Results, Figures S1–S5, and Tables S1–S4 [file mmc1.pdf]

SUPPLEMENTARY INFORMATION

Anhedonia and Anxious Arousal Are Associated With Distinct Expectations About the Statistics of a Volatile Environment

Duda *et al.*

Methods

Sample and Procedure

A sample of 287 participants was recruited via Prolific. Participants were at least 18 years old and residing in the United States. Five attention checks were embedded within the questionnaires (e.g., “fish live in water”). The final sample (*N* = 223) excluded *n* = 4 with incomplete answers, *n* = 31 failing an initial screening attention check, *n* = 19 failing remaining attention checks, *n* = 4 pausing for greater than one minute during a task, and *n* = 6 with inconsistent questionnaire answers (*Careless* R package (1); **Supplemental Results** show findings with inconsistent responders included). The study was initially designed to oversample for internalizing symptoms via a screening survey, but this was deemed unnecessary due to wide symptom variability during piloting and early data collection (see **Main Sample Characteristics**). Participants were paid up to $14 ($5 plus a scaled bonus based on task performance).

Paradigm Validation

To validate the new valence-specific versions of the probabilistic reversal learning (PRL) task, convergent validity was assessed by comparing the following metrics with the established combined-valence task (2–4): 1) number of achieved performance-dependent reversals, 2) portion of the sample achieving reversals, 3) win-switch and lose-stay rates, and 4) associations between task behavior and paranoia.

Generalized Linear Mixed Models (GLMM) Predicting Win-Switch Behavior

Although linear mixed-effects models are generally robust to moderate violations of normality (5), we additionally fit a binomial GLMM with the *lme4* package in R (6) to confirm the association between anxiety and win-switch behavior, given notable right-skewness of win-

switch rates. The counts of win-switches relative to win-stays were utilized as the outcome, with fixed effects of anxiety, valence (gain versus loss), block (pre versus post), and paranoia, and their interactions, and a subject-specific random intercept. To confirm specificity of anxiety findings to win- versus lose-switch behavior, we also fit a binomial GLMM predicting trial-level switch behavior after both wins and losses, with fixed effects of anxiety, task valence, previous trial outcome (win/no-loss or loss/no-win), their interactions, trial number, and paranoia, with a random intercept for subject. Continuous predictors for both models were z-scored to facilitate model convergence. Optimization was performed using the BOBYQA algorithm (7). To further evaluate specificity of altered switch behavior to anxiety, we also ran each model 1) swapping anxious arousal for anhedonia and 2) including both anxious arousal and anhedonia in a single model.

Hierarchical Gaussian Filter (HGF)

We fit a 3-level mean-reverting HGF for the perceptual model. Deck choice (1, 2, or 3) and outcomes (1 = win/no-lose, 0 = no-win/lose) were entered as column vectors with values for each trial. Studies with the original PRL paradigm fit the HGF separately for the first and second half of trials, initializing parameters in the second half with the free parameter estimates from the first half (2–4). However, participants are not informed of the change in task contingencies between blocks and reinitializing the trajectories does not allow for computing a continuous trajectory of posterior mean estimates, so we instead fit a single model across all trials. Given evidence of differences in clinical associations by block in past work (2,4), separate free parameters were estimated within a single model for the first and second half of trials without reinitializing the trajectories, with a single μ^0_3 reflecting initial volatility expectations. We chose

a softmax-μ_3 decision model, which uses third-level μ values to modulate the inverse temperature (2,4,8).

Parameter Recovery

Parameter recovery was performed by refitting the HGF on ten simulations of task data for each agent. Simulated data were generated using the observed parameter estimates together with the perceptual and decision models. Agents' responses were simulated with a softmax function and outcomes generated from a Bernoulli distribution. Correlations between actual and recovered parameter values were computed and a confusion matrix was created to compare model-generated versus observed task behavior.

Optimal Parameter Values

We performed a grid search to test for the μ^0_3 and ω_3 values that produced maximal performance on the task (i.e., greatest number of selections of the highest reward/lowest loss probability decks). We tested 100 evenly spaced values for each parameter from -4 to 4 to cover the range of observed parameters. μ^0_3 and ω_3 were chosen as the focus of the grid search given their demonstrated associations with internalizing symptoms (see **Main Results**) and to reduce the search space. For every combination of parameter values for μ^0_3, block 1 ω_3, and block 2 ω_3 for gain and loss, performance was simulated 10 times (20,000,000 simulations total). All other parameters were set at their median observed value. Performance was computed for each parameter combination, averaged across the ten simulations, and heat maps were created for each pairwise combination of the three parameters. The performance surface of average optimal deck selections across the simulations was smoothed using generalized additive models with the *mgcv* package in RStudio (9) with the basis dimension set to $k = 30$. We defined the optimal parameter region as the parameter combinations producing the highest 5% of smoothed performance. To

determine whether internalizing symptoms were associated with more or less optimal μ^0_3 and ω_3 values, we computed the minimum distance between each subject's observed and optimal-range μ^0_3 and ω_3 values and examined correlations with anxious arousal and anhedonia.

Q-Learning Model

As an alternative approach and to align with past work (2), a simple Q-learning model with a separate learning rate for positive and negative prediction errors was run. The Q-learning rule can be depicted as:

$$Q_{t+1}(s,a) = Q_t(s,a) + \alpha(r_t - Q_t(s,a)),$$

where s is the current state, a is an action, t is the trial, $Q(s,a)$ is the current action value, α is the learning rate computed separately for positive and negative prediction errors, and r_t is the observed reward or loss. The modeling approach was chosen to align with (2).

Model Comparison

Model fit for an HGF with parameters estimated separately by block versus across the full task was compared with summed log-model evidence (LME), which estimates negative variational free energy using the Laplace assumption. LME is recommended for model comparison with the HGF (see TAPAS Manual: https://translationalneuromodeling.github.io/tapas). Model comparison with LME is not possible with the Q-learning model, so BIC was approximated for the best-fitting HGF to facilitate model comparison.

Results

Validating Valence-Separated Probabilistic Reversal Learning Tasks

Behavioral Metrics

Average win-switch rates were .08 and .10 for the gain and loss tasks, respectively, aligning with published metrics from the original combined-valence task (.02-.13 [1]). The average lose-stay rates were .26 and .25, respectively, slightly lower than the original task (.30-

.39). Average performance dependent-reversals (a measure of accuracy) ranged from 5.9-6.0 (original task: 4.9-6.3). In the gain and loss tasks, respectively, 97.8% and 96.0% of the sample achieved reversals (original task: 87.5-100.0%). Associations between task performance (achieved reversals and points earned) and clinical symptoms were not significant (**Table S3**). Together, results indicate similar behavior across task versions and between the new task and the original task, such that clinical differences by valence should not be driven by inconsistencies in task design.

Associations Between Paranoia, Behavior, and Volatility Estimates

Associations between the valence-specific tasks and paranoia also aligned with past studies (2–4,10–12). Specifically, paranoia was associated with a higher win-switch rate, $t(221) = 2.25$, $p = .025$, regardless of task valence, $p = .453$, replicating past work (2–4,11). Paranoia also predicted greater lose-switch behavior, $t(221) = 2.61$, $p = .010$, especially in the gain condition (no-win switch), $t(663) = 2.17$, $p = .031$, which was a novel finding. Paranoid participants exhibited higher equilibrium volatility expectations (m_3), $t(221) = 3.21$, $p = .002$, replicating (4). A paranoia by task valence interaction was also significant, $t(663) = 2.30$, $p = .022$, such that there was a stronger association in the gain versus loss versions. Such valence effects were not possible to examine with the previous version of the task. Paranoia and its interaction with valence were not associated with U-values, *p*s > .200, μ^0_3, *p*s > .494, or ω_3, *p*s > .171.

Sensitivity Analyses

Main Models Incorporating Both Anhedonia and Anxious Arousal

Findings were unchanged in sensitivity analyses covarying the opposing internalizing symptom dimension (i.e., covarying anhedonia when testing associations with anxious arousal and vice versa), while also covarying paranoia. Anxious arousal interacted with task valence to

predict greater win-switch rates, $t(660) = 2.05$, $p = .041$, and greater prior volatility expectations, $t(220) = 2.03$, $p = .043$, in the loss versus gain task. Anhedonia remained linked with lower prior volatility estimates across tasks, $t(219) = -3.21$, $p = .002$, lower U-values in the loss versus gain task, $t(660) = -2.20$, $p = .028$, and a higher meta-volatility learning rate, $t(219) = 2.08$, $p = .039$. The anhedonia, valence, and task block interaction predicting meta-volatility also remained significant, $t(660) = -2.70$, $p = .007$.

GLMMs Predicting Win-Switch Behavior

Anxiety. A binomial GLMM predicting counts of win-switch and win-stay behavior confirmed a valence by anxiety interaction, $b = -0.27$, $SE = 0.05$, $z = -5.59$, $p < .001$, which held when accounting for anhedonia, $p < .001$, indicating a greater influence of anxiety on win-switching in the loss versus gain task. Further, a binomial GLMM predicting trial-level switch probabilities revealed a significant interaction between anxiety, task valence, and previous trial outcome, $b = -0.10$, $SE = 0.04$, $z = -2.25$, $p = .024$, which held in a model controlling for anhedonia, $p = .027$. Simple slopes revealed a significant positive association with anxiety and switch behavior only after no-loss in the aversive version, $b = 0.16$, $SE = 0.08$, $z = 2.15$, $p = .031$, converging with findings with win-switch rates (see **Main Results**).

Anhedonia. In a binomial GLMM predicting counts of win-switch and win-stay behavior, a negative association between anhedonia and switch behavior emerged, $b = -0.22$, $SE = 0.09$, $z = -2.43$, $p = .015$, regardless of task valence, $p = .334$. This effect held when controlling for anxious arousal, $p = .009$. Similarly, in the trial-level binomial GLMM, a significant interaction emerged between outcome (win/no-loss or loss/no-win) and anhedonia predicting switch rates, $b = -.17$, $SE = .03$, $z = -6.60$, $p < .001$, regardless of task valence, $p = .888$. This effect held when controlling for anxiety, $p < .001$. Simple slopes revealed a negative

association between anhedonia and switch behavior specifically after wins (and no-losses), b = -.18, SE = .06, z = -2.82, p = .005.

Accounting for Demographic Variables

Zero-order correlations between age and income and all behavioral and third-level computational parameters were nonsignificant, ps > .08. Participants assigned female at birth showed significantly higher win-switch rates, $t(160) = 2.17$, $p = .03$, and U-values, $t(212) = 2.55$, $p = .012$, on block 1 of the loss task, and higher μ^0_3 estimates on the gain task, $t(217) = 2.53$, $p = .012$. Given significant associations between 1) win-switch rates and anxious arousal, 2) U-values and anhedonia, 3) μ^0_3 and anxious arousal, and 4) μ^0_3 and anhedonia (see **Main Text**), the corresponding mixed-effects models were rerun adding sex assigned at birth as a covariate, which did not meaningfully alter results. Anxious arousal still interacted with valence to predict a higher win-switch rate, $t(657) = 2.80$, $p = .005$, and μ^0_3, $t(219) = 2.24$, $p = .026$, in the loss versus gain tasks. Anhedonia also interacted with valence to predict a lower U-value in the loss versus gain tasks, $t(657) = -2.12$, $p = .035$, and a lower μ^0_3, $t(218) = -2.80$, $p = .006$, regardless of valence, $p = .987$.

Incorporating Inconsistent Responders

The *Careless* package in R was chosen *a priori* to flag inconsistent responders on the questionnaires, leading to the removal of $n = 6$ (1). To confirm that their removal did not meaningfully alter results, we reran all significant models adding back the inconsistent responders. Aligning with the quality-controlled sample, anxious arousal interacted with task valence, $t(675) = 2.75$, $p = .006$, to predict greater win-switch behavior in the loss versus gain conditions. Anxious arousal also interacted with task valence to predict a greater prior on volatility (μ^0_3) in the loss versus gain versions, $t(225) = 2.17$, $p = .031$. As in the smaller sample,

anhedonia interacted with valence to predict lower U-values in the loss versus gain conditions, $t(675) = -2.16$, $p = .031$, predicted a lower overall prior expectation of volatility (μ^0_3), $t(225) = -2.88$, $p = .004$, and interacted with valence and task block to predict meta-volatility (ω_3), $t(675) = -2.74$, $p = .006$.

Q-Learning Model

In mixed-effects models of internalizing symptoms predicting each Q-learning parameter with valence and a subject-specific random intercept, run as a comparison for the HGF, internalizing symptoms and their interactions with valence were not associated with learning rates (α) for positive or negative prediction errors, *p*s ≥ .262, or with the inverse temperature (β), *p*s ≥ .247.

Model Validation

Hierarchical Gaussian Filter

Parameter Recovery. Associations between the original and best-fitting recovered values for each agent were moderate for μ^0_3, *r*s(221) > .31, *p*s < .001, strong for m_3, *r*s(221) > .80, *p*s < .001, and weak for ω_3, *r*s(221) > .14, *p*s < .028. A confusion matrix showed accurate model prediction of participant deck choices (74.1%; **Figure S1**).

Optimal Parameter Values. As depicted in **Figure S2**, lower μ^0_3 values and higher ω_3 values for both task valences generally yielded better performance (although note that when ω_3 approached 4 in block 1, some parameter combinations produced values that violated model assumptions). Anxious arousal was associated with a μ^0_3 estimate that was further from the optimal parameter region in the loss version, $r(221) = .18$, $p = .008$, but not the gain version, $p = .481$. Anhedonia was marginally associated with μ^0_3 values that were closer to the optimal region in the gain condition, $r(221) = -.12$, $p = .066$, and the loss condition, $r(221) = -.12$, $p = .078$.

There were no associations between internalizing symptoms and distance of ω_3 values from the optimal region, *p*s > .184.

Model Comparison

Across task valences, there was greater model evidence for an HGF with separate parameter estimates by block versus single parameter estimates across the task. Specifically, in the split-block versus single-parameter versions of the reward task, summed LMEs were -24,706 versus -25,743, respectively, and for the split-block versus single-parameter versions of the aversive task, LMEs were -24,996 versus -26,070, respectively. The average BIC was lower for the Q-learning model versus winning HGF for both the reward task (BIC_{HGF} = 228.2, BIC_Q = 194.7) and loss task (BIC_{HGF} = 231.8, BIC_Q = 200.2), which was not surprising given the sharp penalty imposed by the BIC for additional free parameters (3 parameters for the Q-learning model, 10 for the split-block HGF). The HGF remained the main focus of the study as it was determined *a priori* to be best-suited to derive volatility estimates. As reported above, the Q-learning model was not related to internalizing symptoms.

Tables

Table S1.

Prior Means and Variances of HGF Perceptual Model Parameters

Parameter	Prior Mean	Prior Variance
$\mu^0{}_3$	1	1
$\sigma^0{}_3$	0.1	1
m_3	1	1
ω_3	0.5	1
κ_2	0.5	1
$\mu^0{}_2$	0	0
$\sigma^0{}_2$	1	0
m_2	0	0
ω_2	-5	16
φ	0.1	0

Note. Prior means and variances for the perceptual model of the mean-reverting three-level hierarchical Gaussian filter. Priors based on the winning model from (8). The parameter φ is in logit space and the initial uncertainties $\sigma^0{}_{2,3}$ and scaling parameter κ_2 are in log space. A prior variance of zero indicates that the parameter was fixed during model estimation. HGF: hierarchical Gaussian filter.

Table S2.

Self-Reported Lifetime Mental Health Diagnoses

**Diagnosis**	*n* (%)
Anxiety Disorder	63 (28.3)
Depressive Disorder	59 (26.5)
Neurodevelopmental Disorder	19 (8.5)
Posttraumatic Stress Disorder	17 (7.6)
Obsessive Compulsive Disorder	11 (4.9)
Bipolar Disorder	9 (4.0)
Personality Disorder	3 (1.3)
Eating Disorder	3 (1.3)
Psychosis	1 (0.4)
Substance Use	1 (0.4)

Note. Participants self-disclosed history of mental health diagnoses. Neurodevelopmental disorders include attention-deficit hyperactivity disorder and autism spectrum disorder (ASD), including two individuals reporting a "suspected" ASD diagnosis. Posttraumatic stress disorder (PTSD) includes individuals self-reporting complex PTSD. The bipolar category includes an individual reporting cyclothymia and another noting "mild" symptoms. Categories may include both current and remitted diagnoses.

Table S3.

Zero-Order Correlations Between Clinical Symptoms and Task Performance

Measure	Achieved Reversals (Gain)	Achieved Reversals (Loss)	Points Earned
Paranoia	-.10	-.09	-.06
Anxious Arousal	-.10	-.12	-.09
Anhedonia	.02	.09	.06
General Distress	.01	.06	.07

Note. Achieved reversals are an index of accuracy, as performance-dependent reversals were triggered after selecting the best deck on nine of the last ten trials. Points earned also index accuracy, as selecting the best deck should yield a higher chance of wins/no-losses. No correlations between clinical symptoms and performance were significant, *p*s $> .05$.

Table S4.

Zero-Order Correlations Between Internalizing Symptoms and Second-Level HGF Parameters

Parameter	κ_2				ω_2			
Task Valence	Gain		Loss		Gain		Loss	
Block	1	2	1	2	1	2	1	2
Internalizing Dimension								
Anxious Arousal	-.07	.01	-.12	.05	-.03	-.03	-.14*	-.10
Anhedonia	-.03	.11	-.01	.04	-.01	.02	.02	-.01
General Distress	-.08	.13	-.02	-.03	-.05	.02	-.02	-.03

Note. Zero-order correlations between internalizing symptoms and second-level parameters derived from a hierarchical Gaussian filter fit to behavior on two probabilistic reversal learning tasks (gain and loss). Clinical symptoms were measured with the Mood and Anxiety Symptom Questionnaire-30-Item Adaptation (13). Anxious arousal was negatively associated with tonic volatility, ω_2 (which governs how quickly beliefs about deck contingencies change over time) during early trials of the loss task (block 1 ω_2), $r(221) = -.14$, $p = .034$; however, associations between anxiety, valence, block, and their interactions predicting ω_2 in a mixed-effects model were not significant, *p*s $\geq .053$. HGF: hierarchical Gaussian filter, κ_2: a coupling parameter between the second and third level of the HGF, ω_2: tonic volatility, * $p < .05$.

Figures

Figure S1.

Recovered versus Actual Third-Level Parameters

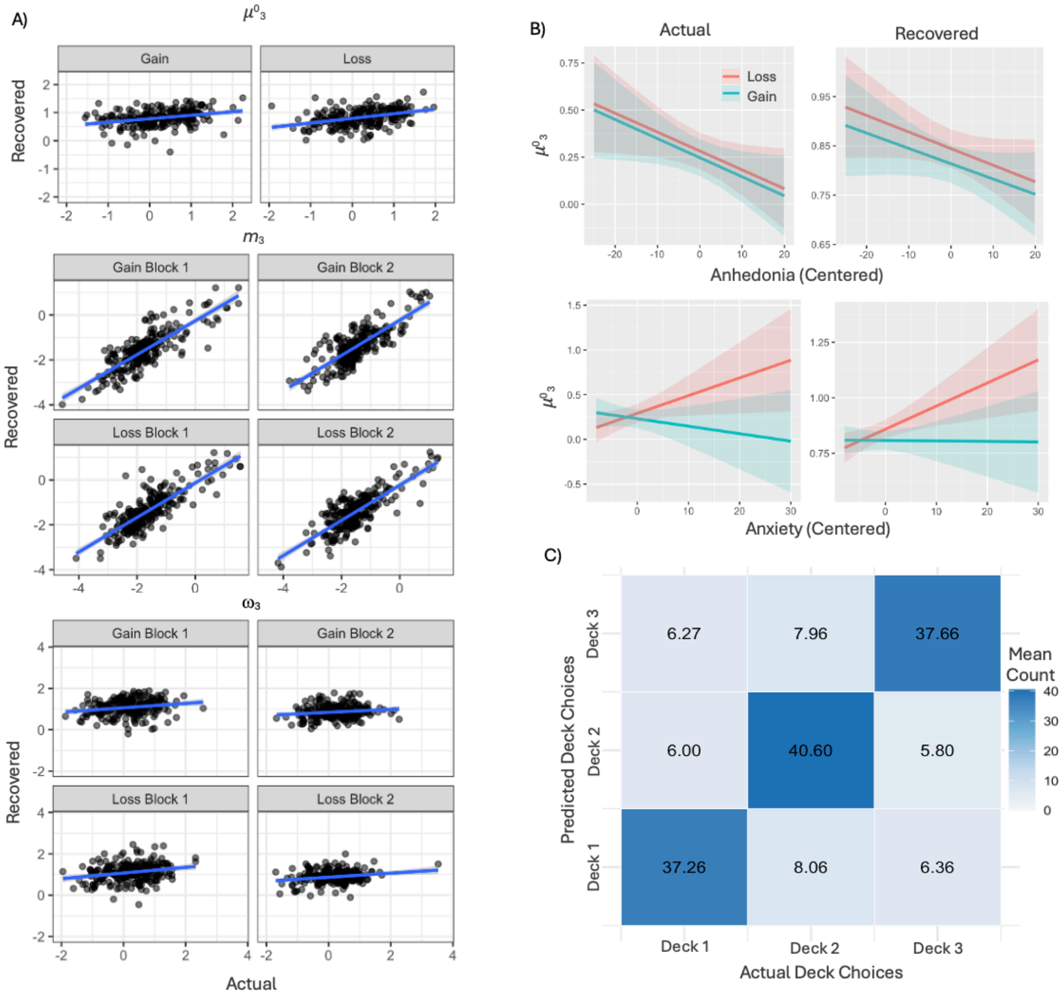

Note. Recovered versus actual parameters. Parameter recovery was performed by refitting the HGF on ten iterations of simulated task data for each agent. A) Correlations between the actual and best-fitting recovered parameters were moderate for μ^0_3, rs(221) > .31, ps < .001, strong for equilibrium volatility (m_3), rs(221) > .80, p < .001, and weak for meta-volatility (ω_3), rs(221) > .14, ps < .028. B) A mixed-effects model showed that anhedonia was negatively associated with the recovered μ^0_3, $t(219) = -2.35$, $p = .020$, controlling for paranoia and task valence, aligning with the observed results. The interaction between anxiety and valence predicting the recovered μ^0_3 was also significant, $t(219) = -2.09$, $p = .038$. C) A confusion matrix depicting counts of observed versus model-generated deck selections averaged across participants revealed accurate model prediction of participant behavior. The model effectively predicted 75.2% of deck 1 selections, 71.7% of deck 2 selections, and 75.6% of deck 3 selections.

Figure S2.

Range of μ^0_3 and ω_3 Values and Simulated Task Performance for A) Gain and B) Loss

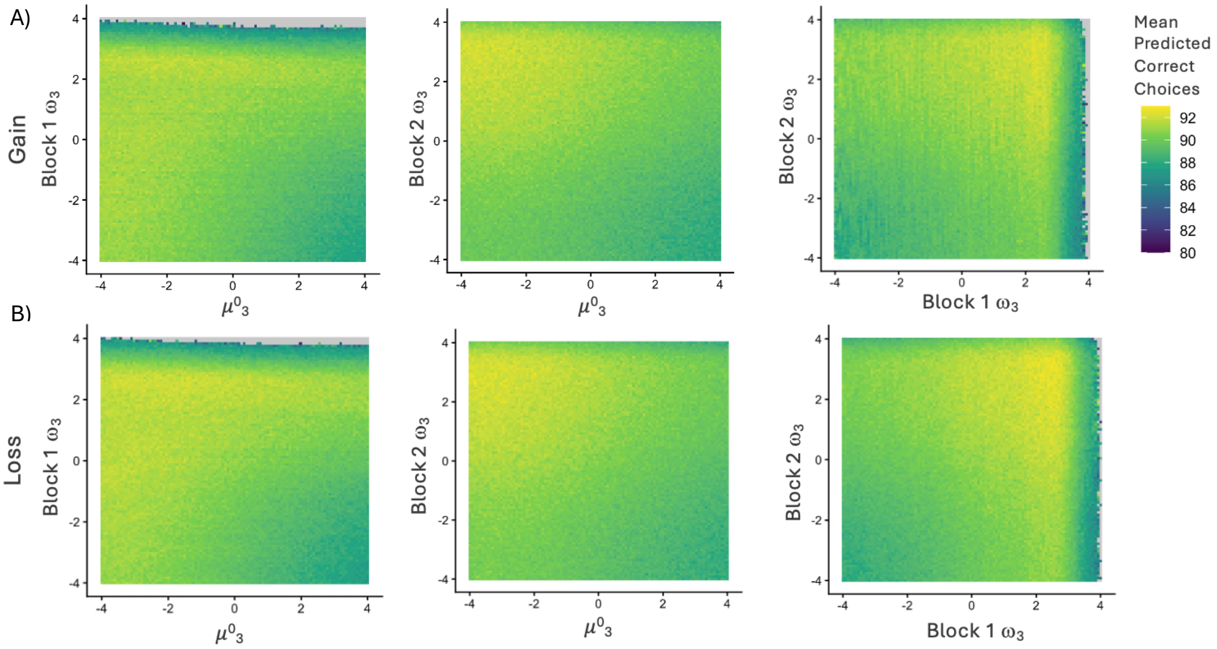

Note. Heat maps depict results of grid search for pairs of μ^0_3 and ω_3 values and simulated task performance (number of best deck selections) for the A) gain task and B) loss task. Performance was simulated 10 times for each combination of 100 evenly spaced parameter values from -4 to 4 for μ^0_3, block 1 ω_3, and block 2 ω_3, and averaged across the simulations. All other parameters were fixed to their median observed values. Parameter combinations that violated model assumptions in at least one simulation are shown in grey. In general, lower μ^0_3 and higher ω_3 values produced better task performance (although block 1 ω_3 parameters approaching 4 produced simulations that violated model assumptions in both tasks).

Figure S3.

Task Behavior and A) Anxiety, B) General Distress, and C) Anhedonia

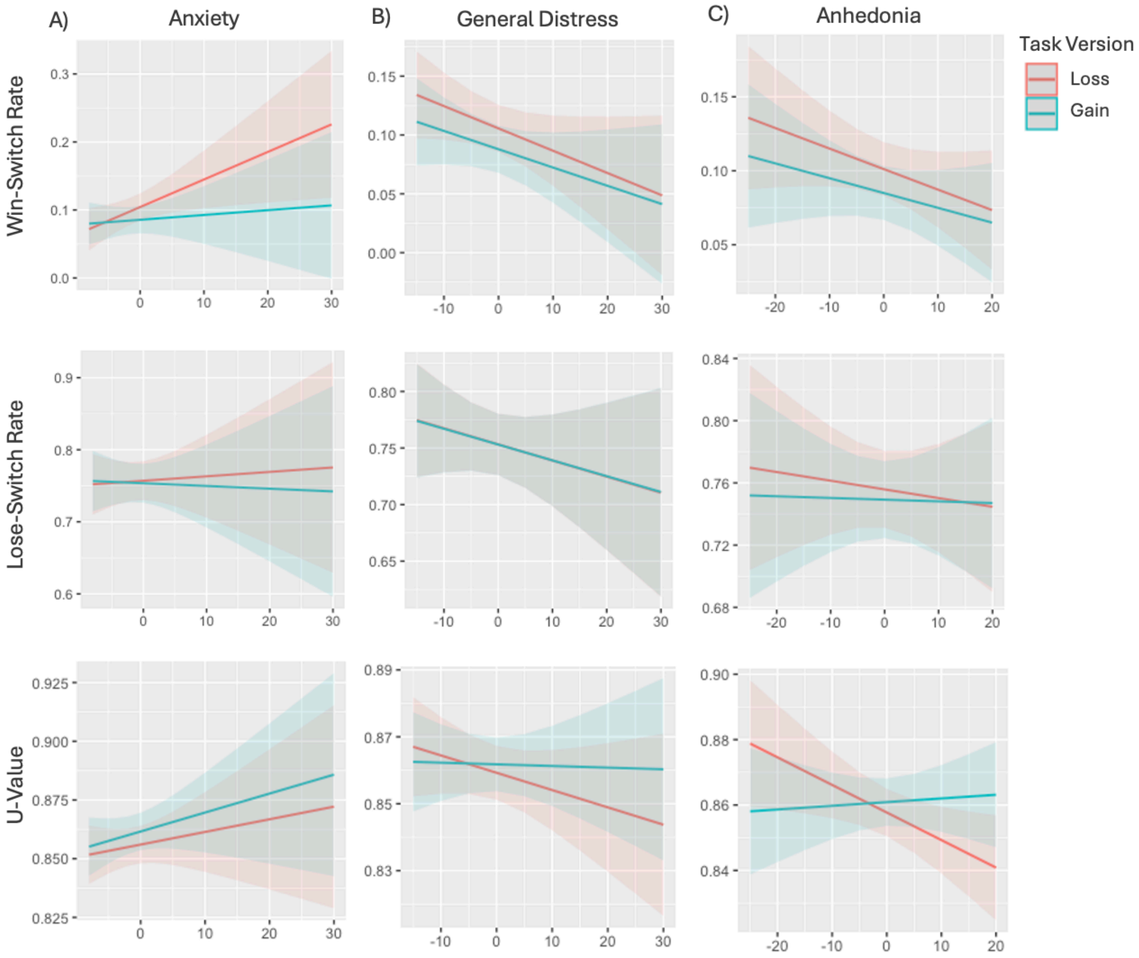

Note. Marginal means from mixed-effects models predicting win-switch rates, lose-switch rates and U-values by task block, task valence (gain versus loss), and clinical dimensions (A. anxious arousal, B. general distress, or C. anhedonia), with a subject-specific random intercept, controlling for paranoia. Anxious arousal, anhedonia, and general distress were measured with the Mood and Anxiety Symptom Questionnaire-30-Item Adaptation (13) and mean-centered prior to analyses.

Figure S4.

Prior Volatility Expectations and A) Anhedonia, B) General Distress, and C) Anxious Arousal

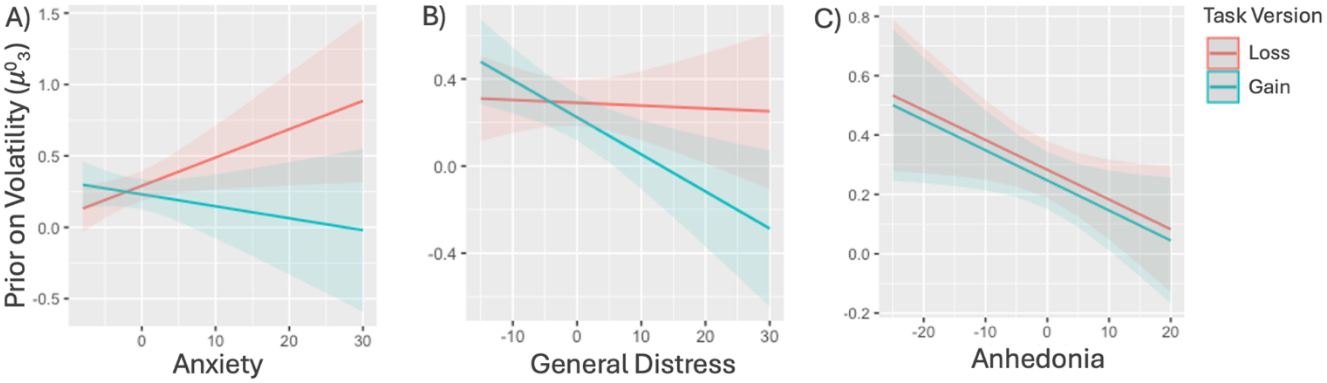

Note. Marginal means from clinical dimensions predicting prior beliefs about volatility (μ^0_3) derived from a 3-level hierarchical Gaussian filter fit to behavior on two probabilistic reversal learning tasks. Clinical symptoms were measured with the Mood and Anxiety Symptom Questionnaire-30-Item Adaptation (13). Depicted results are from linear mixed-effects models predicting μ^0_3 by task valence and A) anxious arousal, B) general distress, or C) anhedonia, controlling for paranoia, with a random intercept for subject.

Figure S5.

Median Trial-Level μ_3 Values for High versus Low Anxious Arousal and Anhedonia

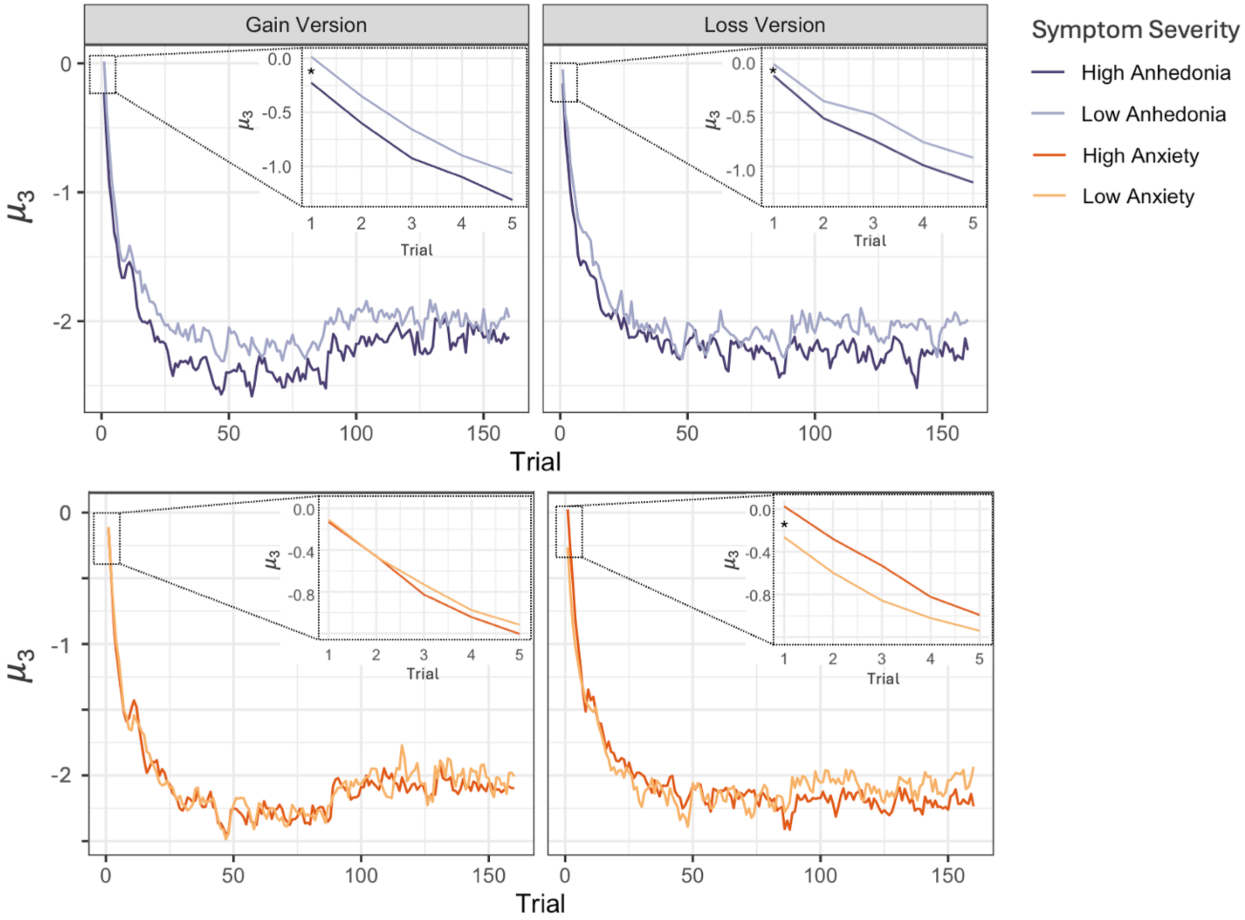

Note. Median trial-level μ_3 values for the gain and loss versions of a probabilistic reversal learning paradigm by clinical symptom severity. Anxious arousal and anhedonia were measured with the Mood and Anxiety Symptom Questionnaire-30-Item Adaptation (13) and separated into high versus low groups based on a median split for visualization purposes only (internalizing symptoms were treated dimensionally for all analyses). * indicates that the corresponding clinical variable (anhedonia or anxiety) significantly predicted μ^0_3 in the dimensional analyses. Anxious arousal was associated with an elevated initial expectation of volatility (μ^0_3) in the loss versus gain versions of the task, $t(219) = 2.24$, $p = .026$, whereas anhedonia predicted a reduced initial expectation of volatility, $t(219) = -2.90$, $p = .004$, regardless of task valence, $p = .987$. Equilibrium volatility expectations (m_3) across the full task were not significantly related to internalizing symptoms, $ps > .05$.

References

1. Yentes R, Wilhelm F. Careless: Procedures for computing indices of careless responding. 2023.

2. Reed EJ, Uddenberg S, Suthaharan P, Mathys CD, Taylor JR, Groman SM, et al. Paranoia as a deficit in non-social belief updating. Schoenbaum G, de Lange FP, Schoenbaum G, editors. eLife. 2020 May 26;9:e56345. doi:10.7554/eLife.56345

3. Suthaharan P, Reed EJ, Leptourgos P, Kenney JG, Uddenberg S, Mathys CD, et al. Paranoia and belief updating during the COVID-19 crisis. Nat Hum Behav. 2021 Sep;5(9):1190–202. doi:10.1038/s41562-021-01176-8

4. Suthaharan P, Thompson SL, Rossi-Goldthorpe RA, Rudebeck PH, Walton ME, Chakraborty S, et al. Lesions to the mediodorsal thalamus, but not orbitofrontal cortex, enhance volatility beliefs linked to paranoia. Cell Rep. 2024 Jun 25;43(6). doi:10.1016/j.celrep.2024.114355 PubMed PMID: 38870010.

5. Schielzeth H, Dingemanse NJ, Nakagawa S, Westneat DF, Allegue H, Teplitsky C, et al. Robustness of linear mixed-effects models to violations of distributional assumptions. Methods Ecol Evol. 2020 Sep 1;11(9):1141–52. doi:10.1111/2041-210X.13434

6. Bates D, Mächler M, Bolker BM, Walker SC. Fitting linear mixed-effects models using lme4. J Stat Softw. 2015 Oct 7;67(1):1–48. doi:10.18637/JSS.V067.I01

7. Powell MJD. The BOBYQA algorithm for bound constrained optimization without derivatives [Technical report]. University of Cambridge; 2009.

8. Cole DM, Diaconescu AO, Pfeiffer UJ, Brodersen KH, Mathys CD, Julkowski D, et al. Atypical processing of uncertainty in individuals at risk for psychosis. NeuroImage Clin. 2020 Jan 1;26:102239. doi:10.1016/j.nicl.2020.102239

9. Wood SN. Fast Stable Restricted Maximum Likelihood and Marginal Likelihood Estimation of Semiparametric Generalized Linear Models. J R Stat Soc Ser B Stat Methodol. 2011 Jan 1;73(1):3–36. doi:10.1111/j.1467-9868.2010.00749.x

10. Rossi-Goldthorpe R, Silverstein SM, Gold JM, Schiffman J, Waltz JA, Williams TF, et al. Different learning aberrations relate to delusion-like beliefs with different contents. Brain. 2024 Aug 1;147(8):2854–66. doi:10.1093/brain/awae122

11. Sheffield JM, Suthaharan P, Leptourgos P, Corlett PR. Belief Updating and Paranoia in Individuals With Schizophrenia. Biol Psychiatry Cogn Neurosci Neuroimaging. 2022 Nov 1;7(11):1149–57. doi:10.1016/j.bpsc.2022.03.013

12. Suthaharan P, Corlett PR. Assumed shared belief about conspiracy theories in social networks protects paranoid individuals against distress. Sci Rep. 2023 Apr 13;13(1):6084. doi:10.1038/s41598-023-33305-w

13. Wardenaar KJ, van Veen T, Giltay EJ, de Beurs E, Penninx BWJH, Zitman FG. Development and validation of a 30-item short adaptation of the Mood and Anxiety Symptoms Questionnaire (MASQ). Psychiatry Res. 2010 Aug 30;179(1):101–6. doi:10.1016/j.psychres.2009.03.005
